# COVID-19 and Acute Aortic Syndromes: Understanding the Dynamic Interplay

**DOI:** 10.1055/s-0041-1735979

**Published:** 2021-10-08

**Authors:** Francesca Terzi, Rossella Fattori, Marco Di Eusanio

**Affiliations:** 1 Azienda Ospedaliera Ospedali Riuniti Marche Nord, Pesaro PU, Italy; 2 Dipartimento di Cardiochirurgia, Centro di Scienze Cardiovascolari Ospedale Lancisi, Università Politecnica delle Marche, Ancona AN, Italy

We would like to thank Mori and colleagues 1 for their interest in our article “Intramural hematoma as unexpected complication of COVID 19,” recently published in *AORTA* . 2 They raise some stimulating issues with regard to the possible relationship between coronavirus disease 2019 (COVID-19) inflammation and acute aortic syndromes, as presumed in our patient. The contribution of inflammation to the incidence of cardiovascular disease has been increasingly recognized, embracing the widest spectrum of vascular pathologies, from myocardial infarction to abdominal aortic aneurysm. Currently, multiple cytokines, including interleukins, interferon, the tumor necrosis factor superfamily, colony-stimulating factor, chemotactic factor, and growth factor, have been demonstrated to play a critical role in aortic dissection. 3 In particular, a cytokine storm has been recognized in the development of acute aortic syndromes, as the interaction and the systemic imbalance between pro- and anti-inflammatory action plays a major role in its formation.

Vascular damage has been previously identified in other inflammatory diseases such as rheumatoid arthritis, psoriasis, and lupus erythematosus. 4 While we acknowledge that pathological examination of the resected aorta would have better supported our hypothesis, it remains conceivable that the potent inflammatory stimulus of COVID-19 disease, affecting multiple organ systems, might have a role even in the development of acute aortic syndromes. Mori and colleagues, 1 who also reported their surgical experience 5 in a case of intramural hematoma and COVID-19 disease, argued that in our patient this association could be allocated to a simple coincidence. Indeed, a simple coincidence could always be advocated in concomitant diseases. Several cases of stroke, myocardial infarction, and myocarditis have been reported as complications of COVID-19 outbreak. Undeniably, our patient did not have any risk factors such as hypertension or family history; the aorta was normal in diameter, supporting the absence of any preexisting subclinical aortic disease.

Interestingly, recent studies 6 7 during the COVID-19 pandemic highlight a decline in hospital presentation for acute conditions such as acute coronary or aortic syndromes along with a 10-fold increase in the number of at-home deaths. These data, rather a true change in epidemiology, probably reflect a lesser attention by general practitioners to patient symptoms or patient fear of contracting COVID-19 if presenting to the emergency department.

Unexpected consequences of COVID-19 pandemic may add some further concern on health system vulnerabilities, causing delay in diagnosis and management of oncologic or cardiovascular patients with long-term catastrophic impact on mortality.
